# Optogenetic innovations in stem cell therapy: Illuminating the path for transplanted cells after spinal cord injury

**DOI:** 10.4103/NRR.NRR-D-25-01817

**Published:** 2026-01-27

**Authors:** María del Mar Sánchez-Martín, Victoria Moreno-Manzano, Esther Giraldo

**Affiliations:** Department of Neurosurgery, School of Medicine, Stanford University, Palo Alto, CA, USA; Neuronal and Tissue Regeneration Laboratory, Prince Felipe Research Center (CIPF), Valencia, Spain; Department of Biotechnology, within the Interuniversity Research Institute for Molecular Recognition and Technology Development (idm UPV-UV), Universitat Politècnica de València, Valencia, Spain

Unleashing the regenerative capacity of the central nervous system following spinal cord injury (SCI) remains a challenging frontier in the field. Despite extensive efforts, the complex pathophysiology of SCI, characterized not only by the immediate damage of the primary injury but also by the subsequent cascades of events over time, presents significant obstacles to meaningful functional recovery (Ahuja et al., 2017). These processes vary significantly among SCI patients, further hindering the effectiveness of existing therapeutic interventions. The incidence of SCI varies worldwide, with approximately 25 new cases per million individuals annually (Ahuja et al., 2017), leading to long-lasting physical, psychological, and social consequences.

In recent years, numerous therapeutic approaches have emerged to address various barriers to neural regeneration, including inflammatory signaling, immune cell infiltration, and the inhibitory extracellular matrix. However, it has become apparent that no single approach sufficiently addresses the multifaceted nature of SCI. Most current interventions are focused on managing the devastating consequences after SCI, with limited effectiveness, and they often fail to tackle the root cause of paralysis: the loss of neuronal connections and compromised spinal cord architecture.

Cell-based therapies, particularly those involving neural progenitor cells (NPCs), emerged as effective strategies for SCI treatment, demonstrating both neuroprotective and neuroregenerative capacities, addressing multiple aspects of the injury’s pathophysiology (Hosseini et al., 2024). NPCs have the potential to: (1) replace lost neurons and glial cells, providing the building blocks necessary for improving connectivity; (2) secrete neurotrophic factors; (3) modulate neuroinflammation; and (4) remodel the lesion environment to create a permissive extracellular matrix (Fischer et al., 2020; Hosseini et al., 2024). NPC transplantation has demonstrated anatomical and functional integration with host neural circuits, forming new synaptic connections beyond the injury site. They support the regeneration, extension, and remyelination of injured axons required for voluntary movement, such as the corticospinal tract. However, they face several limitations, such as (1) limited cell survival, with a significant initial loss of grafted cells, (2) the challenge of selecting the appropriate cell type for differentiation into functionally relevant cells, and (3) limited locomotor recovery (Fischer et al., 2020).

The translational potential of cell therapy faces several additional hurdles, including the need for larger quantities of NPCs to establish robust transplants in the human spinal cord, ensuring cell survival, and the requirement for NPCs to cover longer axonal growth distances for effective neural relay formation in human injuries and for achieving meaningful recovery. Nevertheless, the translation of NPC therapies into clinical settings has been actively pursued (Hosseini et al., 2024). Phase I clinical research using human central nervous system-derived NSCs has demonstrated safety, feasibility, and benefits in functional recovery for patients with chronic cervical SCI. Furthermore, multiple clinical trials using NPCs obtained from induced pluripotent stem cells have been developed. Although autologous transplantation would be the best option to avoid rejection, allogenic transplantation is the most practical way to have the treatment available when needed, because obtaining the patient’s induced pluripotent stem cells would be expensive and time-consuming.

In recent years, the use of neuromodulation techniques with stem cell transplant has garnered increasing attention for its potential to enhance the neuroregenerative capacities of transplanted cells (Fischer et al., 2020). With the emergence of optogenetic approaches, it has become feasible to achieve spatiotemporally precise control over cellular signaling. Neuroscientists have long envisioned optogenetics as a means to modulate neuronal activity and study circuit dynamics (Geng et al., 2023). However, it also presents a promising therapeutic strategy for central nervous system injuries, serving as a tool for the targeted stimulation of transplanted cells after SCI, paving the way for innovative approaches in stem cell therapy.

Accordingly, we previously demonstrated how blue-light stimulation of NPCs engineered to ectopically express the excitatory light-sensitive protein channelrhodopsin-2 (NPC-ChR2) prompted an increase in proliferation and differentiation into oligodendrocytes and neurons with increased branching and axonal length, even in the presence of axonal inhibitory drugs. Furthermore, optogenetic modulation polarizes NPC-derived astrocytes from a pro-inflammatory phenotype to a pro-regenerative/anti-inflammatory phenotype, highlighting the potential to increase neuroregeneration and improve cell therapy outcomes after SCI (Giraldo et al., 2020).

**Figure 1:**
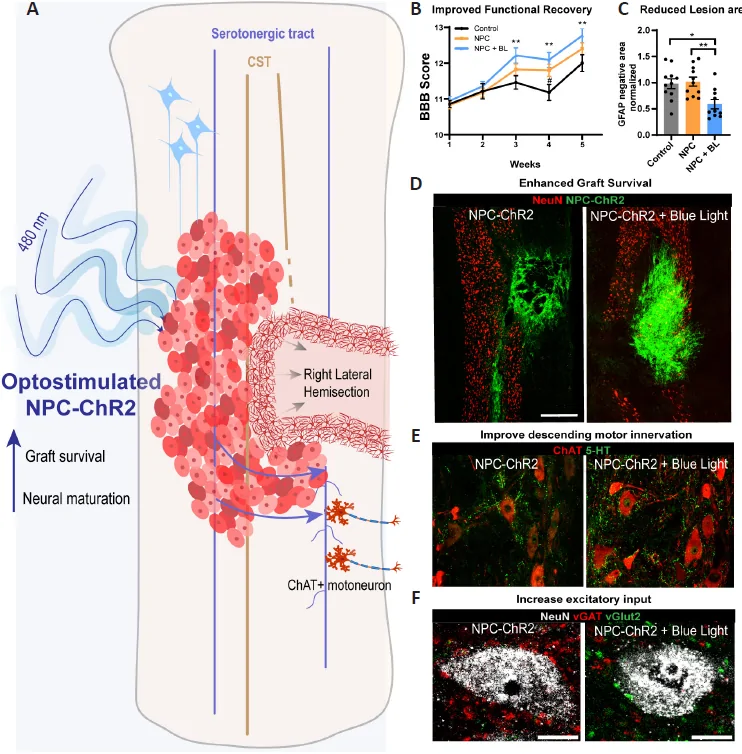
Transplantation of optogenetically stimulated NPC-ChR2 cells improves motor recovery after spinal cord injury. (A) Illustrative overview of NPC-ChR2 findings following optogenetic stimulation. (B) BBB locomotor rating scale shows improved functional recovery after optogenetic stimulation of NPC-ChR2 over 5 weeks in NPC + BL (blue), NPC (orange), and control (black) rats. Data evaluated by two-way analysis of variance followed by Tukey’s multiple comparisons test (control group *n* = 14; NPC *n* = 17; NPC + BL *n* = 18). **P < 0.01. (C) GFAP-negative injured area is reduced after optogenetic stimulation of NPC-ChR2. Data are expressed as mean ± SEM. *P < 0.05, **P < 0.01, ****P < 0.0001 (ordinary one-way analysis of variance and Sidak’s multiple comparisons tests). (D) We found the survival of a significantly higher number of NPC-ChR2 after optogenetic stimulation compared with the non-stimulated transplanted group (NPC: 350.8 ± 106.8; NPC + BL: 783.6 ± 88.58 positive cells per mm^2^; **P = 0.0054). Horizontal sections of NPC-ChR2 (green) and NeuN-positive neurons (red) near the injury site, 4 weeks post-transplantation, show enhanced survival of optogenetic stimulated graft. Scale bar: 500 μm. (E) Higher preserved 5-HT fibers (green) in optogenetic in optogenetically stimulated NPC-ChR2 rats significantly improved the serotonergic innervation of ChAT-positive motoneurons (red) contralesional caudal to the injury compared with non-stimulated NPC-ChR2 (*P = 0.037) and control rats (*P = 0.028) (control: 52.11 ± 8.38; NPC: 53.90 ± 3.69; NPC + BL: 80.38 ± 3.42). Scale bar: 50 μm. (F) Optogenetically stimulated NPC-ChR2 transplanted rats displayed a significantly enhanced excitatory (vGlut2, green)/inhibitory (vGAT, red) ratio of host neurons (NeuN, red) contralesional to the lesion compared with both NPC (****P < 0.0001) and control rats (**P = 0.0052) (control: 35.90 ± 4.46; NPC: 29.70 ± 2.84; NPC + BL: 52.47 ± 3.55). Scale bar: 50 μm. Reprinted from Sánchez-Martín et al. (2025). BBB: Basso, Beattie, and Bresnahan; BL: blue-light; ChR2: channelrhodopsin-2; GFAP: glial fibrillary acidic protein; NPC1β neural progenitor cell; vGAT: vesicular gamma-aminobutyric acid transmitter; vGlut2: vesicular glutamate transporter 2.

Recently, we also demonstrated that optogenetic stimulation of transplanted NPC-ChR2 in a rat model of incomplete thoracic SCI significantly enhances motor recovery with improved outcomes than the non-stimulated NPCs (Sánchez-Martín et al., 2025; **Figure 1A** and **B**). Achieving meaningful functional recovery after SCI is crucial to defining the potential of treatment, since it demonstrates the significance of the subsequent observed anatomical outcomes. Among others, daily optogenetic stimulation of transplanted NPC-ChR2 reduces the astrocytic reactivity, the injured area volume, and enhances graft survival and neuronal maturation (**Figure 1C** and **D**). This provides a permissive and non-inhibitory environment with efficacy for improving host circuit plasticity and descending motor tract regrowth and preservation. The stimulation allowed for the preservation of a higher number of descending propiospinal neurons and a higher innervation of 5-hydroxytryptamine fibers from the reticulospinal tract to choline acetyltransferase-positive motoneurons below the injury (**Figure 1E**). The rewiring of specific types of neurons at the thoracic level after SCI has been reported to be essential for walking, in addition to preservation and/or regeneration of other supraspinal descending neurons, including the corticospinal tract or reticulospinal tract, which also remain critical for restoring coordinated function after SCI (Sánchez-Martín et al., 2025).

Furthermore, optogenetic stimulation of transplanted NPC-ChR2 increased the expression of the vesicular glutamate transporter vesicular glutamate transporter 2 within host neurons (**Figure 1F**). Increasing the excitatory input to the structurally preserved circuit significantly impacts the recovery, due to the dramatic loss of supraspinal inputs (mainly excitatory) after SCI. Thus, we hypothesized that the sustained stimulation of NPC-ChR2 would contribute to reducing the injury’s progressive expansion, promoting early maturation, and accelerating primary injury resolution, contributing to higher spared tissue preservation involving a paracrine effect surrounding the engrafted area (Sánchez-Martín et al., 2025).

Our own results align with other studies that show how optogenetic stimulation *in vitro* induces neurogenesis in neural stem cells and promotes survival. The rise of the intracellular Ca^2+^ levels has been described as the main player leading these cellular signatures. Intracellular Ca^2+^ dependent signaling includes the phosphorylation of cyclic adenosine monophosphate response element-binding protein through the Ca^2+^/calmodulin kinase pathway, and extracellular signal-regulated kinase pathways have been described as the main players of the modulation of neural fate following optogenetic stimulation (Geng et al., 2023). Interestingly, recent advancements in optogenetic tools have focused on enhancing cyclic adenosine monophosphate signaling in neurons to facilitate recovery after SCI. Indeed, we recently demonstrated that the photoactivatable adenylate cyclase in corticospinal neurons has been shown to enhance locomotion through a cortical rerouting pathway via the serotonergic descending tract after SCI (Martínez-Rojas et al., 2025).

Other studies have also focused their effort on innovative optogenetic stimulation for stem cell therapies, reporting results that are consistent with our findings. Previous studies in stroke models have shown how optogenetic stimulation of transplanted NPCs derived from human induced pluripotent stem cells results in differential gene expression, with upregulation of transcripts involved in membrane-associated ion channel functions, neuronal differentiation and plasticity, and downregulation of pro-inflammatory genes (Daadi et al., 2016). Using chemogenetics, Okano’s team revealed that activated iNSCs improved cell function, increased neuron-to-neuron interactions *in vitro* (Kawai et al., 2021). Increased calcium influx by chemogenetic stimulation of hM3Dq-expressing neural cells has been reported to increase expression of immediate-early genes, including *bdnf*, *jun*, *atf3*, *fos*, and *mcl1*, involved in transcriptional programs linked to survival and neuronal plasticity. Thus, this stimulation mechanism, similarly to optogenetics approaches, is not only cell-autonomous but may also influence neighboring cells, as supported by existing studies (Daadi et al., 2016). We also evidenced a strong paracrine regulatory effect through the partially transduced NPC population, minimizing the potential risk of phototoxicity, while significantly impacting the surrounding astroglia and microglia, alleviating the secondary damage (Sánchez-Martín et al., 2025). They reported increased expression of genes and proteins related to synapses in the surrounding host tissue, prevented spinal cord atrophy, and improved locomotor function after SCI *in vivo* (Kawai et al., 2021).

It is essential to highlight that optogenetic stimulation has the potential to significantly enhance paracrine signaling from NPC-ChR2 to neighboring non-transfected cells (Sánchez-Martín et al., 2025). To date, the minimum percentage of NPC-ChR2-transduced cells required to elicit functional benefits has not been clearly defined in the literature. In our previous study (Sánchez-Martín et al., 2025), we observed that *in vitro* optogenetic stimulation of NPC-ChR2 significantly reduced the expression of *cxcl10*, *tnf-α*, and *il-1*β in astrocytes through a paracrine effect, as measured in the conditioned media used to stimulate the recipient NPCs. These factors are known to play a key role during the secondary damage and inflammatory cytotoxic responses. These findings, in conjunction with previous research, indicate that optogenetic stimulation may enhance paracrine signaling from NPC-ChR2 to the non-transfected neighboring cells (Daadi et al., 2016; Sánchez-Martín et al., 2025). This finding opens a new perspective that may reduce costs and minimize interventions. Nevertheless, further investigation is required for key unresolved questions, such as the duration of AAV9-mediated ChR2 expression and the optimal time frame and stimulation procedure for achieving maximum therapeutic benefits.

Similarly, optogenetic innovations in cell therapy face some extra translational limitations, including the feasibility of viral vector-based transduction in humans, the efficacy and stability over time of opsin viral transduction, the complexity of delivery of blue light through device implantation, and limited blue-light penetration. Despite these challenges, optogenetics remains at the forefront due to its fast-acting (milliseconds) mechanism, providing a more precise stimulation compared to chemogenetic approaches (from minutes to hours) with less chemically-induced cytotoxicity (Bansal et al., 2023).

Recent advancements in the field could facilitate the translational path. Non-viral vectors, such as dendrimers, cationic lipids, magnetic nanoparticles, and hydrogels, can be an alternative to carry opsins-dependent gene regulation, also for *in vivo* application. However, their relatively low transfection efficiency generally requires repeated administration, introducing additional interventions (Bansal et al., 2023). Nevertheless, clinical trials are now assessing the safety and efficacy of viral opsin-based therapies for treating neurological deficits in patients. The clinical use of adenoviral carriers for optogenetics has been approved by the United States Food and Drug Administration. Currently, viral vector-based transduction is the most commonly used method to deliver foreign genes to a specific tissue in mammals and is already being used in humans to treat vision disorders (NCT02556736, NCT03326336, and NC04278131). Furthermore, this technology is being explored in other areas like severe epilepsy and Parkinson’s disease, where opto-deep brain stimulation offers targeted neuronal stimulation but would require the insertion of an optical probe to deliver light to a large number of cells, of which only a desirable fraction would respond.

Substantial efforts have been invested in the discovery and engineering of optogenetics with higher sensitivity to red-shifted wavelengths of light, associated with reduced potential for tissue photodamage and greater tissue penetration. Several red-shifted photoresponsive proteins have been described, including ReaChR2, ChrimsonR, Chrmine, and Chronos, each with distinct wavelength sensitivity, kinetics, and photocurrent profiles. Activation of ReaChR2 through non-invasive transcranial stimulation has been successfully used to evoke neuronal responses in the motor cortex (Bansal et al., 2023). Recently developed new step-function opsins with ultra-high light sensitivity, based on SFO (ChR2 C128S/D156A) and incorporating T159C mutations, have demonstrated extremely high light sensitivity. These opsins can undergo photoactivation even under conditions of significantly attenuated light power.

Light delivery remains a technical challenge; however, designs for flexible, implantable, wireless, and non-invasive optogenetic devices are underway to target the nervous system while reducing mechanical stress. Among wireless platforms, microLED-based systems are considered leading candidates and should ideally be flexible, ultrathin, lightweight, durable, low-heating, and easy to implant. Wireless optogenetics has also been integrated with pharmacological delivery, as demonstrated by wireless optofluidic probes incorporating a uLED array, enabling multiplexed fluid and drug delivery. Additionally, transcranial optogenetic stimulation techniques using near-infrared excitation have recently been developed to enable extracranial neuromodulation (Bansal et al., 2023). An alternative to device implantation is the use of optical transducers, such as upconversion nanoparticles, which convert near-infrared light into visible wavelengths to activate well-established photoresponsive proteins.

The current studies show the transformative potential of optogenetics in NPC-based therapies for SCI. Future investigations will be essential to assess the translational feasibility of this innovative approach, not only for SCI but also for other central nervous system injuries. There is a compelling need for multidisciplinary cooperation to illuminate the pathway toward more efficient stem cell therapies in clinical practice.

*The work is supported by the projects PID2021-1243590B-I100 and PID2024-159513OB-I00 (to VMM) from the Agencia Española de Investigación, MICIN/AEI/10.13039/501100011033/FEDER, EU, “Una manera de hacer Europa” and CIPROM/2022/25 (to VMM) from Generalitat Valenciana (to VMM).*
